# Study on a Novel Biodegradable and Antibacterial Fe-Based Alloy Prepared by Microwave Sintering

**DOI:** 10.3390/ma14143784

**Published:** 2021-07-06

**Authors:** Bin Deng, Yingxue Guo, Ming-Chun Zhao, Qing-Fen Li, Bin Ma, Bohua Duan, Dengfeng Yin, Andrej Atrens

**Affiliations:** 1 Research Institute of Automobile Parts Technology, Hunan Institute of Technology, Hengyang 421002, China; 2005001476@hnit.edu.cn; 2 School of Materials Science and Engineering, Central South University, Changsha 410083, China; y.x.guo@csu.edu.cn (Y.G.); duan-bh@csu.edu.cn (B.D.); 3 School of Mechanical Engineering, Hunan Institute of Technology, Hengyang 421002, China; 2011001704@hnit.edu.cn (Q.-F.L.); 2007001529@hnit.edu.cn (B.M.); 4 School of Mechanical and Mining Engineering, The University of Queensland, Brisbane, QLD 4072, Australia; andrejs.atrens@uq.edu.au

**Keywords:** Fe-based alloy, microwave sintering, biodegradation, antibacterial function

## Abstract

This research produced a porous Fe-8 wt.% Cu alloy by microwave sintering in order to achieve (i) an increased biodegradation rate, and (ii) an antibacterial function. The Fe-8Cu alloy had higher density, hardness and degradation rate (about 2 times higher) but smaller and fewer surface pores, compared to the pure Fe. The Fe-8Cu alloy had a strong antibacterial function (the antibacterial rates against *E. coli* were up to 99.9%) and good biocompatibility. This work provides a novel approach of alloy design and processing to develop novel antibacterial Fe-based alloys.

## 1. Introduction

Fe-based alloys are highly attractive for metal medical implants due to their biodegradability, good biocompatibility and excellent mechanical properties [1,2,3,4,5]. However, Fe-based alloys have degradation rates that are too slow in the physiological environment, which limits their clinical applications [6,7]. For example, Kraus et al. [8] found the stent strut of a Fe-based alloy was still relatively complete after 360 days implanted in a blood vessel. Such a slow degradation rate directly affects the physiological reactions between the surrounding biological tissue and the implant, such as protein adsorption, immune system regulation, and tissue rejection reaction [9,10,11]. The degradation rate of Fe-based alloys may be increased by the addition of noble alloying elements to form small precipitates to produce micro-galvanic corrosion with the Fe matrix [12,13]. On the other hand, surgical infection may occur for medical implants [14,15,16]. Avoiding and effectively treating such an infection has become an urgent problem in the medical field, as is the abuse of antibiotics that may lead to the increase of bacterial resistance and thus is not the best way to effectively treat infections. Antibacterial Fe-based alloys as bone implant materials are needed that have an inherent long-term antibacterial effect that depends on their own anti-infection ability. The alloying of Fe with a non-antibiotic antimicrobial element is a possible approach to achieve this inherent long-term antibacterial effect.

Based on the above, it is highly desirable to produce Fe-based alloys containing Cu to achieve the following two purposes: (i) an increased biodegradation rate, and (ii) an Fe-based alloy with an antibacterial function. Cu is an essential trace element to human health, which has many beneficial biological functions [17,18,19]. Cu and Fe have the standard potential of +0.34 V_SHE_ [20] and −0.44 V_SHE_ [5], respectively, which leads to the micro-galvanic corrosion effect between Cu-rich precipitation and Fe matrix in Fe-Cu alloy, increasing the corrosion rate. The release of Cu^2+^ with corrosion progressing can produce the desired antibacterial effect, as was recently confirmed by Guo et al. [21], who used selective laser melting to produce antibacterial Fe-xCu alloys having a satisfactory biodegradation rate compared to pure iron.

Fe-based alloys with a porous structure can have a higher biodegradation rate due to the penetration of the body fluid into the porous structure. Microwave sintering is a new rapid sintering method that was successfully used to produce porous alloys in biomedical application [22,23]. Compared with conventional sintering, microwave sintering has higher microwave heating efficiency and more uniform heating, which can shorten the sintering time, enhance the diffusion of elements, and thus improve the mechanical properties [24]. Therefore, it is expected that microwave sintering would produce Fe-Cu alloys with relatively high mechanical strength and a significantly increased degradation rate. Accordingly good antibacterial function is expected due to Cu^2+^ release. The Fe-Cu alloys would become a type of potential novel antibacterial implant materials for clinical application. However, to our best knowledge, never before microwave sintering has been used to prepare Fe-Cu alloys for antibacterial bone implant applications.

This work used Cu powder and Fe powder to prepare high-performance antibacterial Fe-Cu alloys by microwave sintering, and investigated their microstructure, hardness, biodegradation, antibacterial function and cytocompatibility.

## 2. Experiment Methods

### 2.1. Sample Preparation

**Figure 1:**
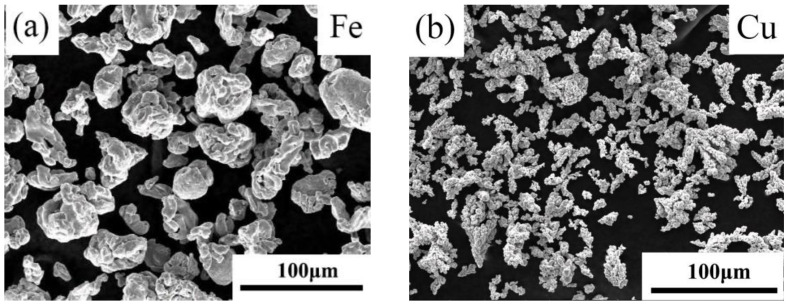

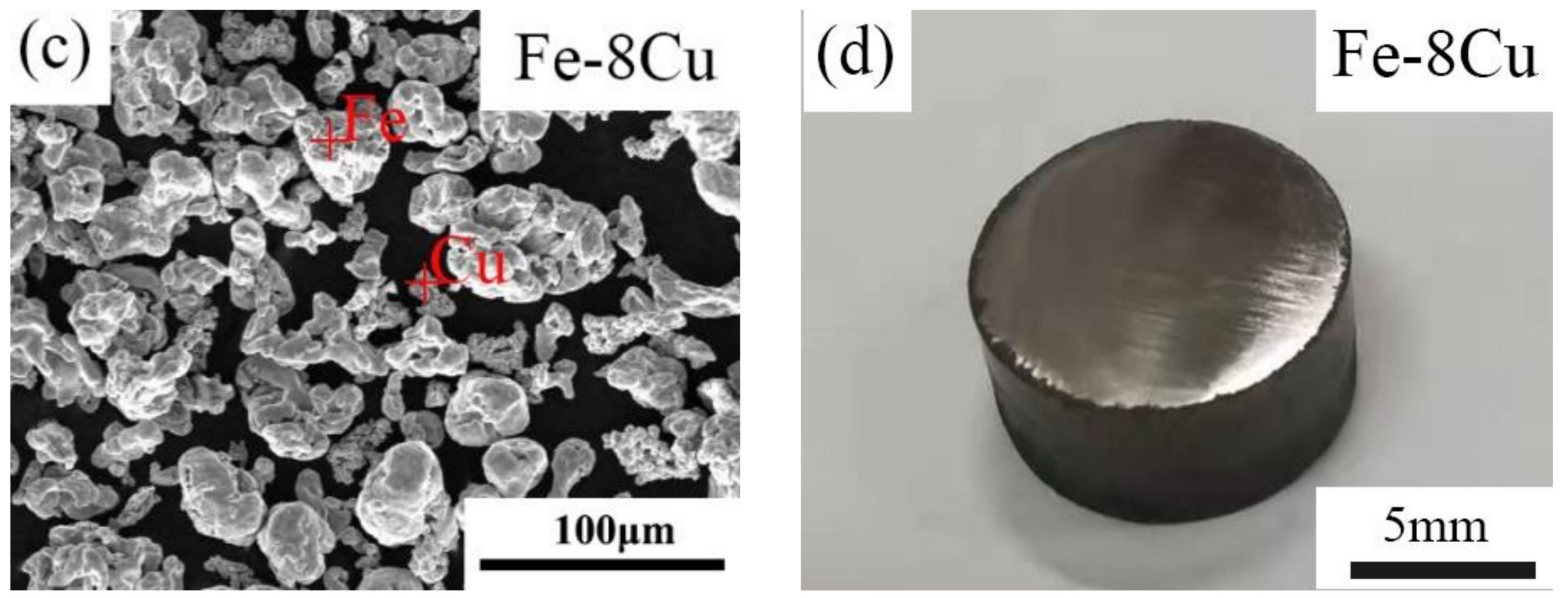
The SEM morphology of the Fe powders (**a**), Cu powders (**b**), Fe-8Cu powder mixture (**c**); and a representative digital macrograph of a microwave-sintered Fe-8Cu sample (**d**).

Figure 1 shows the SEM morphology of the Fe powders (a), Cu powders (b), Fe-8Cu powder mixture (c); and a representative digital macrograph of a microwave-sintered Fe-8Cu sample (d). Fe powders (particle size distribution of ≤70 μm) (Figure 1a) and Cu powders (particle size distribution of ≤50 μm) (Figure 1b) were used as raw materials, with purities of 99.9%, and were purchased from Changsha Tid Metal Materials Co. Ltd., Changsha, China. Based on previous process optimization [21], Fe powders were mixed with 8 wt.% Cu powders (designated as Fe-8Cu). Figure 1c shows the morphology of the mixed Fe-8Cu powders, which were prepared by ball-milling (YXQM-0.4L, Changsha Miqi Instrument equipment Co. Ltd., Changsha, China) with a rotational speed of 300 rpm for 3 h. Ball to powder ratio was 5:1.

The mechanically mixed powders were uniaxially compacted at 600 MPa for 2 min to make cylindrical samples (height about 5 mm, diameter about 10 mm). The mold was demolded at a rate of 12 mm/min using zinc stearate as a mold wall lubricant. The resultant cylindrical samples were placed into a ceramic crucible with SiC powders as microwave acceptor. This ceramic crucible was placed inside a glass tube of a 2.45 GHz 4 kW microwave sintering furnace (HY-ZG1512). The cylindrical samples were heated at a rate of 20 °C/min to 1100 °C via microwave heating under the protection of an Ar and H_2_ atmosphere, sintered for 10 min, and furnace-cooled to room temperature. The temperature upon sintering was monitored using an infrared pyrometer. A representative digital macrograph of a microwave-sintered Fe-8Cu specimen with ϕ10 mm × 5 mm is shown in Figure 1d. The microwave-sintered sample had a shiny surface and apparently small pores, which indicated that the quality of microwave-sintered sample was satisfied in the macroscopic state. The pure Fe samples were the control group and were produced with the same sintering parameters.

### 2.2. Microstructure and Hardness

The microstructures were characterized using an optical microscope (OM, ZEISS Axio Vert Al) and a scanning electron microscope (SEM, ZEISS EVO-18) with an energy dispersive spectrometer (EDS, OXFORD X-MaxN) after grinding using the SiC emery papers from 400 to 2000 grit, and mirror-polishing using a 1.5 μm diamond paste. The densities of the microwave-sintered samples were measured using the Archimedes method [25]. Hardness was determined on the polished surface by a HM-2T Vickers micro-indenter with a 4.903 N test load and 10 s duration. Ten points were selected randomly for each specimen to obtain an average hardness.

### 2.3. Degradation Behavior

The biodegradation behaviors were evaluated using electrochemical tests in Hanks’ Solution (containing 8.00 g/L NaCl, 0.40 g/L KCl, 0.10 g/L MgCl_2_·6H_2_O, 0.35 g/L NaHCO_3_, 0.10 g/L MgSO_4_·7H_2_O, 0.14 g/L CaCl_2_, 0.12 g/L Na_2_HPO_4_·12H_2_O, 0.06 g/L KH_2_PO_4_ and 1.00 g/L glucose) with a pH of 7.6. Potentiodynamic polarization curves were measured with a scan rate of 1 mV s^−1^ using an electrochemical workstation (Metrohm Autolab PGSTAT128N) with a three-electrode cell consisting of the specimen as the working electrode, a saturated calomel reference electrode (SCE) and a counter electrode. The working electrode was the specimen with a 1 cm^2^ area.

The corrosion rate, *P*_i_ (mm/year), was evaluated from the corrosion current density, *i*_corr_ (μA cm^−2^) using Equation (1) [21].

(1) $$P_{i}=1.337\times 10^{-2}i_{corr}$$

Samples for the immersion tests were exposed for 30 d in the Hanks’ solution, which was refreshed every 24 h. After the immersion test, the surface corrosion products were removed by a solution with 2 g antimony oxide, 5 g stannous chloride and 100 mL hydrochloric acid. The degradation rate, *P*_w_ (mm/year), was evaluated using Eqatuion (2) [21].

(2) $$P_{i}=22.615\left(W_{i}-W_{f}\right)$$

where *W*_i_ was the initial sample mass (g) before immersion tests, and *W*_f_ was the final sample mass (g) after immersion tests.

### 2.4. Antibacterial Properties and Cytocompatibility

The antibacterial performance of the sintered cylindrical samples was evaluated by the *E. coli* (ATCC 25922) bacterial counting method. Bacterial suspensions at a 10^5^ CFU mL^−1^ concentration were put into the samples extracts and cultured for 24 h. The obtained co-cultured bacterial suspensions were then diluted into 10^3^ CFU mL^−1^. 100 μL was uniformly plated on AGAR plates and cultured for 24 h. The viable bacteria were quantified. The plate colony-counting method was used for counting. The active bacteria were counted in accordance with National Standard of China (GB/T 4789.2-2010). The antibacterial rate was calculated using the following Equation (3).

(3) $$K=\frac{\alpha_{0}-\alpha_{t}}{\alpha_{0}}\times 100\%$$

where *K* was the sample antibacterial rate, $\alpha_{0\;}$ was the bacterial colony number of the control group, and correspondingly, $\alpha_{t}$ was the sample bacterial colony number.

The cytocompatibility of the sintered cylindrical samples was studied by MG63 human osteosarcoma cells (American Type Culture Collection, Manassas, USA) as a model. The MG63 cells were pre-cultured in Dulbecco’s modified eagle medium (DMEM) [26]. The samples were cultured for 24 h in DMEM using 3 cm^2^ mL^−1^. The supernatant was withdrawn, and the filter membrane was used to prepare the extracts. The extracts were refrigerated at 4 °C before the next tests. For the cell activity tests, MG63 cells were cultured in DMEM 24-well plates. The extracts replaced culture solution of every well after 4 h. The cells were cultured for 3 d, rinsed gently by phosphate buffered solution, stained for 20 min by Ethidium homodimer-1/Calcein-AM reagent. The cells were fixed on a glass slide, and observed by a fluorescence microscope (Olympus BX60, Tokyo, Japan). For cell proliferation tests, the MG63 cells were incubated in 96-well plates with about 2 × 10^3^ cells per 100 μL DMEM. The culture solutions of every well were replaced after 24 h by the sample extracts. After culturing for 1 d and 3 d, 10 μL CCK-8 solutions were instilled to every well, and those MG63 cells were further incubated at 37 °C for 2 h. Optical densities (OD) were measured by a micro-plate reader (thermo scientific multiskan go, USA) at a 450 nm wavelength. The viability of MG63 cells was calculated by Equation (4).

(4) $$Cell\;Viability=\left(\frac{OD_{\mathrm{sample}}}{OD_{\mathrm{negative}}}\right)\times 100\%$$

where *OD*_sample_ is the optical densities of the sample, and *OD*_negative_ is the negative control.

## 3. Results and Discussion

### 3.1. Microstructures and Hardness

The hardness and density of the microwave-sintered samples are presented in Table 1. The Vickers hardness of the microwave-sintered pure Fe was ~101 HV, much higher than that of conventional cast Fe of ~63 HV [3]. The hardness of microwave-sintered Fe-8Cu was higher at ~127 HV. This indicated that (i) microwave sintering markedly increased hardness, and (ii) the incorporation of Cu into pure-iron further effectively increased hardness. Hardening by Cu was attributed to the introduction of particle strengthening. Compared with pure Fe, the density of Fe-8Cu was only slightly increased, indicating that the effect of alloying Cu on the density was negligible. The density of the microwave-sintered pure Fe was ~6.85 g/cm^3^ and that of the microwave-sintered Fe-8Cu was ~6.94 g/cm^3^. The measured density implied that the microwave-sintered pure Fe and Fe-Cu samples had the porous structure.

**Figure 2:**
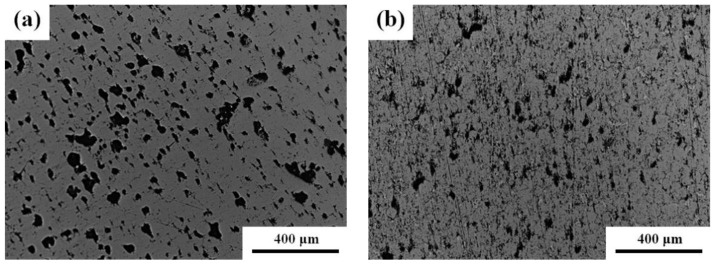
The optical micrographs of (**a**) microwave-sintered pure Fe and (**b**) Fe-8Cu samples.

Figure 2 presents optical micrographs of the microwave-sintered pure Fe and Fe-8Cu. The surface had many randomly distributed pores attributed to (i) power gap (inter-particle pore), (ii) impurity evaporation, and (iii) Kirkendall pores [27]. There were many large pores (~115 μm) and small pores (~11 μm) distributed randomly on the surface of the microwave-sintered pure Fe. However, there were few large pores (~65 μm) and small pores (~5 μm) distributed on the surface of the microwave-sintered Fe-8Cu. The pore size and number for the microwave-sintered Fe-8Cu were smaller than for the microwave-sintered pure Fe attributed to one or more of the following mechanisms: (i) Cu melted and diffused into the boundaries of the Fe particles to fill the pores, (ii) the volume shrinkage increased, and (iii) some pores merged with other pores or disappeared due to the closure of the sintering neck.

**Figure 3:**
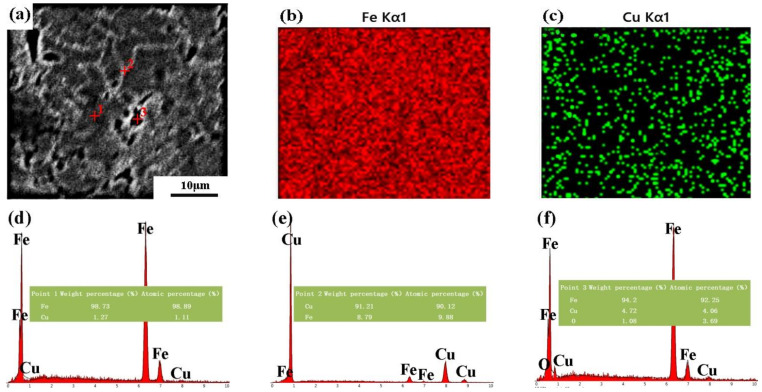
The SEM micrographs of the microwave-sintered Fe-8Cu sample (**a**), EDS maps of Fe element (**b**) and Cu element (**c**) corresponding to (**a**). EDS spectrums corresponding to point 1 (**d**), point 2 (**e**) and point 3 (**f**).

Figure 3 shows the SEM-EDS results of the microwave-sintered Fe-8Cu. Figure 3a shows that the microstructure had a nearly continuous white network, indicating that the Cu had melted and diffused into the Fe-particle boundaries forming Fe-Cu solid solutions and Cu-rich particles. The EDS results confirmed that the microstructure contained a Fe-Cu solid solution and a Cu-rich phase as shown in Figure 3b–f. The Cu was uniformly distributed in the microstructure as revealed by the EDS maps of Fe (Figure 3b) and Cu (Figure 3c) corresponding to Figure 3a. The compositions of the different sites in Figure 3a were analyzed by EDS shown in Figure 3d–f. The EDS results indicated that the gray locations (like point 1) was mainly composed of Fe (Figure 3d), corresponding to the Fe matrix. The white areas (like point 2) were mainly composed of Cu and contained a small amount of Fe (Figure 3e), i.e., the Cu-rich phase. Point 3 represented holes on the surface and the corresponding EDS spectrum (Figure 3f) showed that the hole contained a little oxygen. The presence of oxygen on the surface of the sample meant that the sample was oxidized during sintering. From the oxygen content on the energy spectrum of point 3, it could be determined qualitatively to only be little oxidized.

As stated above, after Cu was alloyed into Fe by microwave sintering, a small quantity of Cu were dissolved into the Fe matrix, and the remaining Cu formed a Cu-rich phase distributed along the Fe-particles boundaries.

### 3.2. Degradation Behavior

**Figure 4:**
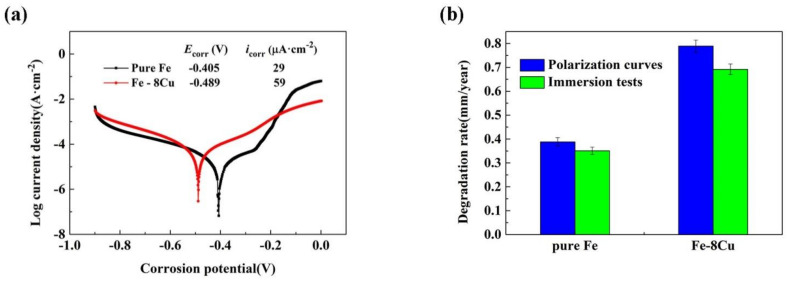
Degradation properties of microwave-sintered pure Fe and Fe-8Cu samples: (**a**) polarization curves; (**b**) calculated degradation rates.

Figure 4a shows the polarization curves of the microwave-sintered pure Fe and Fe-8Cu. The corrosion potential (*E*_corr_) and corrosion current density (*i*_corr_) were evaluated by the Tafel extrapolation method [28,29]. The polarization curve of the microwave-sintered pure Fe had an obvious current platform in the anode region, as shown in Figure 4a, which indicated that the pseudo-passivation film might be formed on the surface, affecting the corresponding degradation rate [21]. However, for the microwave-sintered Fe-8Cu samples, the anode branch current of polarization curve increased steadily with increasing potential.

The degradation rates were evaluated according to the polarization curves and the immersion tests, and are presented in Figure 4b. The polarization curves indicated that the degradation rate of pure Fe was 0.39 mm/year and that of Fe-8Cu was 0.79 mm/year. The immersion tests indicated that the degradation rate of pure Fe was 0.35 mm/year, while that of Fe-8Cu was 0.69 mm/year. The degradation rate of microwave-sintered Fe-8Cu was significantly greater than that of pure Fe (about twice), indicating that Cu increased the corrosion rate.

This study, combined with previous studies [30,31], found that the higher corrosion rate of microwave-sintered Fe-Cu was related to the porous structure and the precipitates. The porous Fe-Cu samples had an actual contact surface area larger than that of the dense Fe-Cu samples, and crevice corrosion may also occur in the porous structure. Moreover, the porous structure of microwave-sintered Fe-Cu may facilitate the release of copper ions and improve the antibacterial properties. Furthermore, the second phase precipitated in the microwave-sintered Fe-8Cu samples was a Cu-rich phase, which produced galvanic corrosion acceleration, caused by the difference of the standard potential between the Fe matrix (−0.44 V_SHE_) and the Cu-rich phase (+0.34 V_SHE_). The increase of corrosion rate of the microwave-sintered Fe-Cu sample is beneficial to the release of Cu ions, resulting in better bactericidal performance.

### 3.3. Antibacterial Properties and Cytocompatibility

**Figure 5:**
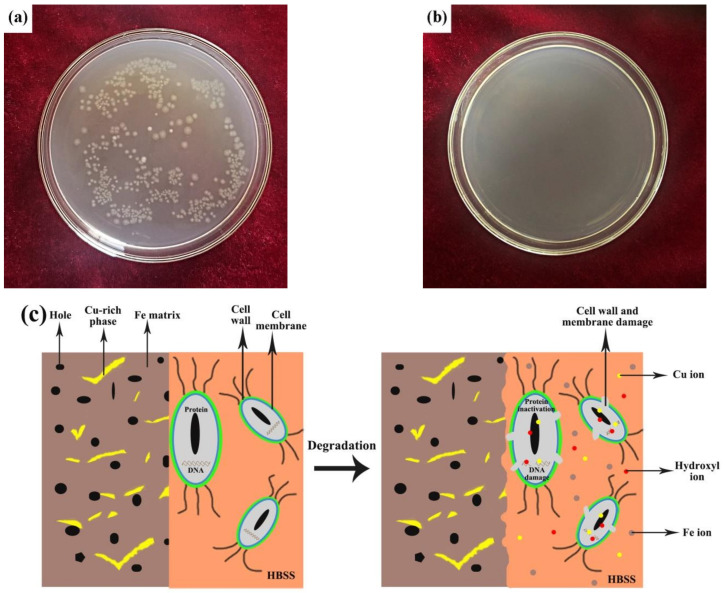
The representative image for the *E. coli* colonies incubated for 24 h on surface of the microwave-sintered pure Fe (**a**) and Fe-8Cu (**b**). The antibacterial mechanism for microwave-sintered Fe-Cu samples in simulated physiological environment (**c**).

Figure 5a,b shows representative images for the *E. coli* colonies that were incubated for 24 h on the surface of the microwave-sintered pure Fe (the control group) and Fe-8Cu samples at 37 ± 0.5 °C, respectively. There were a large number of *E. coli* colonies on the microwave-sintered pure Fe, but there were no *E. coli* colonies on the microwave-sintered Fe-8Cu samples. The antibacterial rates of the Cu ions released from the microwave-sintered Fe-8Cu samples were up to 99.9% effective against *E. coli*, which indicated that the microwave-sintered Fe-8Cu samples had excellent antibacterial properties.

The antibacterial mechanism for microwave-sintered Fe-Cu samples in simulated physiological environment is summarized in Figure 5c. The structure and phase composition of the microwave-sintered Fe-Cu sample were the main factors causing the good antibacterial effects. The porous microwave-sintered Fe-Cu samples greatly increased the contact area between the bacteria and the samples, and also facilitated the release of Cu ions and improved antibacterial properties. In addition, the porous structure could also cause perforation, deformation and damage of the bacterial cell membranes, resulting in bacterial cell death. Moreover, the Cu-rich phase formed during the microwave sintering ensured the good antibacterial properties of the Fe-Cu samples. Redox reactions occurred when the Cu-rich phase on the surface of microwave-sintered Fe-Cu samples contacted the solution. These redox reactions were mainly caused by the two factors, Cl^-^ and Fe^3+^ ions, which could promote the release of Cu ions [20]. The Cl^-^ ions were in the solution. The Fe^3+^ (+0.77 V_SHE_) ions were produced by the galvanic reaction of Fe (−0.44 V_SHE_ V) and Cu (+0.34 V_SHE_). The released Cu ions contacted the bacteria on the surface of microwave-sintered Fe-Cu samples. Cu ions extracted electrons from bacteria and damaged the permeability of bacterial cell membrane, resulting in the loss of bacterial cytoplasm [16]. Cu ions and hydroxyl ions could interact with the thiol groups of the bacterial proteins, which could induce the rupture of the ionic bonds of proteins and lead to the inactivation of the proteins. Furthermore, Cu ions and hydroxyl ions could bind to bacterial DNA, which could inhibit its replication [32,33,34]. Therefore, Cu ion could kill bacteria through destroying the cell walls and membranes, deactivating the activity of proteins and inhibiting DNA replication. Hence, the microwave-sintered Fe-Cu samples could have good antibacterial function because of the porous structure and the action of Cu ion released.

**Figure 6:**
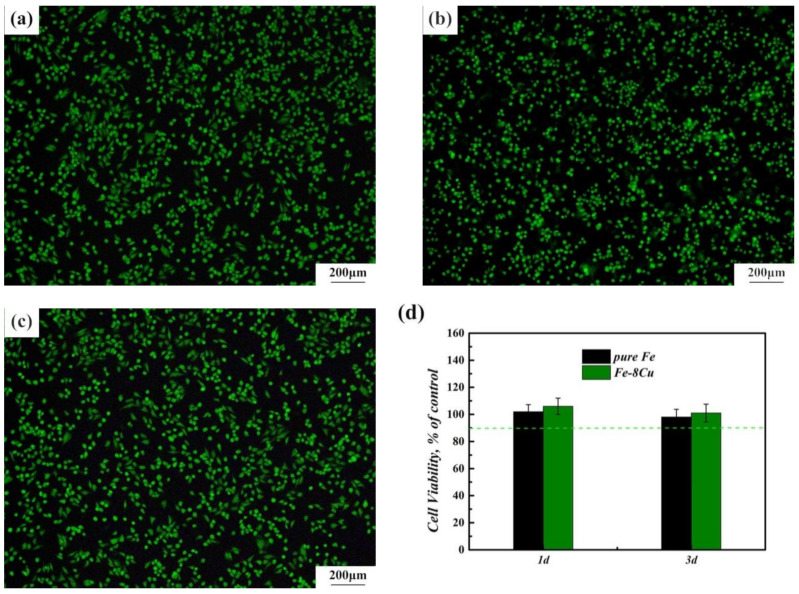
The fluorescence morphologies of MG63 cells after 3 d culture in DMEM (**a**), pure Fe (**b**) and Fe-8Cu (**c**) extracts. The cell viability of MG63 cells in extracts of microwave-sintered samples after culturing 1d and 3d (**d**).

Figure 6a–c shows the fluorescence morphologies of MG63 cells after being cultured for 3 days. Alive cells were green and dead cells were red. The fluorescence morphology of MG63 cells corresponding to the microwave-sintered pure Fe and Fe-8Cu samples showed no significant difference in cell densities compared with the control group. There were large numbers of living cells in the fluorescence morphology images, indicating normal cell proliferation, i.e., the microwave-sintered pure Fe and Fe-8Cu samples had no cytotoxicity. Cell viability is an important indicator for cytocompatibility. A cell viability 75% higher in control group indicates that the materials was not cytotoxic based on ISO 10993-5. The cell viabilities of the MG63 cells were 90% higher in the control group in Figure 6d, indicating that the microwave-sintered pure Fe and Fe-8Cu samples were not cytotoxic. Therefore, the microwave-sintered pure Fe and Fe-8Cu samples had good cytocompatibility.

## 4. Conclusions

This research produced a Fe-Cu alloy by microwave sintering in order to achieve an increased biodegradation rate and an antibacterial function. The following conclusions were obtained. Bulk Fe-8Cu alloy and bulk pure Fe were successfully prepared by microwave sintering. Alloying with Cu has an evident influence on density, hardness, and biodegradation rate, that is, the Fe-8Cu alloy had higher density, hardness and degradation rate (about 2 times higher) but smaller and fewer surface pores, compared to the pure Fe. Furthermore, the Fe-8Cu alloy had a strong antibacterial function (the antibacterial rates against *E. coli* were up to 99.9%) and good biocompatibility. This work provides a novel approach of alloy design and processing to develop novel antibacterial Fe-based alloys.

## Figures and Tables

**Table 1:** Density and hardness of the microwave-sintered pure Fe and Fe-8Cu.

	Pure Fe	Fe-8Cu
Density (g/cm^3^)	6.85 ± 0.003	6.94 ± 0.002
Relative density (%)	87.04 ± 0.04	87.30±0.06
Hardness (HV)	101 ± 2	127 ± 1

## Data Availability

Data sharing not applicable.
